# Pressure‐Induced Drift Artifacts in Stretchable Liquid Metal ThinFilm Electrocardiogram Electrodes

**DOI:** 10.1002/advs.76002

**Published:** 2026-07-02

**Authors:** Ding Li, Zi‐Gan Xu, Zhi‐Kang Chen, Shuo‐Yan Xu, Jia‐Yi Cui, Si‐Yuan Wo, Zi‐Xu Wang, Yi‐Kun Liu, Jia‐Ju Yin, Hou‐Fang Liu, Xiao‐Ming Wu, Lu‐Qi Tao, Yi Yang, Tian‐Ling Ren

**Affiliations:** ^1^ School of Integrated Circuit Tsinghua University Beijing China; ^2^ Beijing National Research Center for Information Science and Technology (BNRist) Tsinghua University Beijing China

**Keywords:** electrocardiogram, liquid metal, motion artifacts, strain sensors, stretchable, thin‐film electrodes

## Abstract

Reliably monitoring epidermal electrophysiological signals with precision is essential for advanced healthcare systems and next‐generation human‐machine interfaces. Although stretchable thin‐film electrodes have shown promise for accurate electrocardiogram signal acquisition, the issue of pressure‐induced drift artifacts remains largely overlooked. We developed a stretchable LM thin‐film electrode integrated with an LM strain sensor to quantitatively investigate the drift artifact alongside skin deformation simultaneously and in situ. While most existing research focuses on strain‐induced drift, our findings reveal that the pressure‐induced drift artifact is more significant in stretchable LM electrodes. This work further emphasizes the limitations of the skin‐electrode impedance model in explaining the pressure‐induced drift, and confirms that its primary origin lies in skin potential change. Based on this, we validated an adaptive filtering method using the noise signal reconstructed from strain sensor data to calibrate pressure‐induced drifts. Compared to traditional static filtering methods, it demonstrates superior performance in suppressing irregular pressure‐induced drift artifacts

## Graphical abstract

A stretchable LM electrode integrated with a strain sensor enables in situ quantitative investigation of drift artifact and skin deformation. This reveals the significance of pressure‐induced drift artifact and its intimate relationship with the skin potential model. To suppress such artifact, an adaptive filtering method is established using the indentation signal reconstructed from the strain sensor.

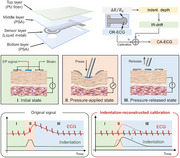

## 1 Introduction

Wearable flexible electronics offer a promising avenue for seamless human‐machine integration, sparking significant interest in developing skin‐like stretchable electronic devices [1, 2, 3, 4]. Among the diverse physiological signals amenable to wearable sensing, epidermal electrophysiological signals play a crucial role in modern medicine as essential indicators and diagnostic tools. For instance, electrocardiogram (ECG) remains the gold standard for non‐invasive cardiovascular disease diagnosis and management [5, 6]. Despite notable progress in structure design [7, 8, 9, 10] and materials [11, 12, 13, 14, 15, 16], motion artifacts in wearable electrophysiological devices remain a significant challenge [17].

The replacement of traditional snap electrodes or suction cup electrodes with stretchable thin‐film electrodes represents a significant technological advancement in wearable ECG signal acquisition. Stretchable thin‐film electrodes conform well to the skin, thereby effectively mitigating motion artifacts caused by electrode‐skin interface mismatch during daily activities, thus enabling precise dynamic signal acquisition [7, 9, 18]. However, pressure‐induced drift in these electrodes had been overlooked since previous studies assumed that the skin is primarily under strain, not compression. In reality, compression of the torso and limbs is ubiquitous in daily life, with limbs accounting for most human‐environment interactions. Examples include pressure from resting arms on a desk, constrictive clothing or accessories, and carrying loads. Therefore, a systematic assessment of electrode performance under pressure and the development of effective calibration methods are crucial for reliable real‐world applications.

**FIGURE 1:**
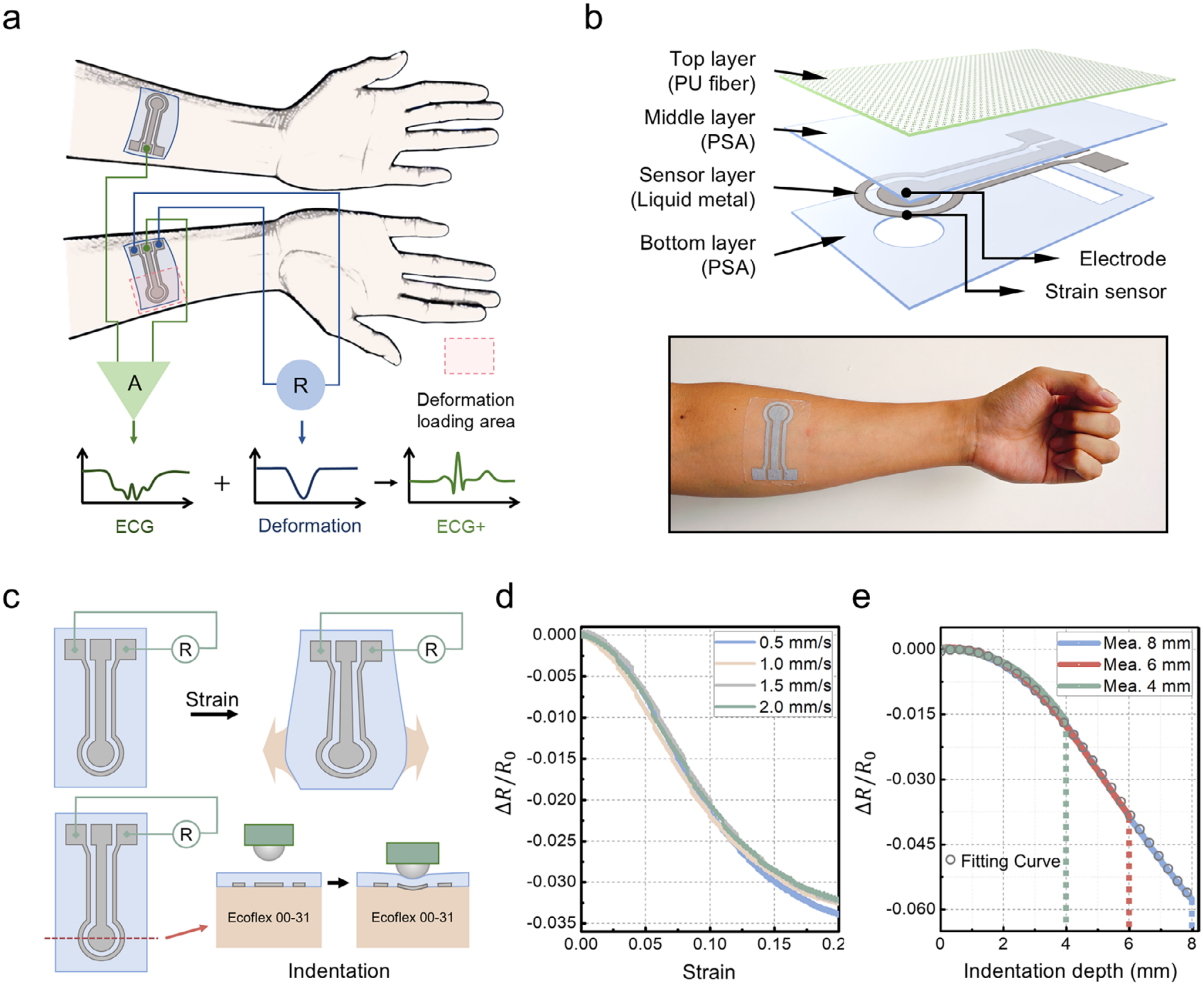
Description of the testing platform. (a) Simultaneously detect the ECG signal and skin deformation, and calculate the calibrated ECG signal (ECG+). (b) Illustrative disassembly view and photograph of the stretchable LM thin‐film electrode. (c) Experimental setup for measuring the responses of the LM strain sensor to strain (upper) and indentation (lower). (d) Resistance change rate versus strain curves under different strain rates. (e) Resistance change rate versus indentation depth curves under different measuring ranges. Hollow circles denote the fitting results.

In this study, we quantitatively investigate the drift artifacts in ECG signals acquired by stretchable liquid‐metal (LM) thin‐film electrodes and their relationship with skin deformation (Figure 1a). Our findings demonstrate that the stretchable LM thin‐film electrode suppresses strain‐induced interference effectively, but experiences significant pressure‐induced drift. Experimental analysis reveals that conventional skin‐electrode impedance models fail to account for this significant disparity. We therefore identify varying skin potential as the primary cause of pressure‐induced drift [19]. Inspired by the impulse response of the first‐order RC circuit and transepidermal potential difference, we developed a method to reconstruct these drift artifacts from indentation depth data. Combined with adaptive filtering, this enables real‐time calibration of electrocardiogram signals under irregular pressure. This method significantly outperforms conventional static calibration, yielding a 50% greater reduction in drift artifacts while also adapting to varying pressure interference.

## 2 Results

### 2.1 Preparation and Validation of the Testing Platform

To enable the concurrent, in situ measurement of epidermal ECG signals and adjacent skin deformation, we developed a stretchable LM thin‐film electrode integrated with a strain sensor (Figure 1b). It comprises a four‐layer structure: the bottom and middle layers consist of pressure‐sensitive adhesive (PSA), serving as the skin adhesion layer and assembly bonding layer respectively; the core sensing layer incorporates liquid metal (gallium‐indium eutectic) with circular stretchable electrodes and surrounding annular strain sensors (Figure S1); and the top layer is a thin electrospun polyurethane (PU) fiber film that functions as a stretchable supporting substrate. The electrode was fabricated via screen printing (Figure S2), and it combines softness (Young's modulus ∼ 96 kPa) with a thin‐film design (thickness 200 µm), enabling firm conformal adhesion to the human skin surface for stable test data acquisition (Figure S3 and Movie S1). Besides, different stretchable LM electrodes were available utilizing different surface modification methods (Figure S4).

This electrode combines excellent ECG signal acquisition with skin deformation detection capability. At low frequencies (≤ 100 Hz), the skin‐electrode impedance and intrinsic impedance of the stretchable LM electrodes exhibited significantly lower than that of commercial button gel electrodes (Figure S5). The annular LM strain sensor around the electrode can effectively respond to both the strain and indentation applied to the electrode. The experimental setup for measuring the stretchable strain sensor's response to strain and indentation is shown in Figure 1c: the strain direction was maintained perpendicular to the signal leads; the indentation was applied using a steel hemispherical body to press downward on the electrode. The response curves under various test conditions in Figure 1d,e demonstrates that variations in strain rate and indentation depth have minimal impact on the response curves (more details see Figure S6). The Fourier numerical fitting approach was utilized to establish the quantitative mapping function between strain/indentation and resistance, resulting in a high degree of accuracy with an R‐squared value of 0.998 when comparing fitted results to experimental data (Figure 1e, details see Methods). For the signal acquisition system of the test platform, ECG signals and strain sensor signals were synchronously acquired by two sets of systems configured for temporal alignment (Figure S7).

### 2.2 Pressure‐Induced Drift Artifacts in Stretchable LM Thin‐Film Electrodes

**FIGURE 2:**
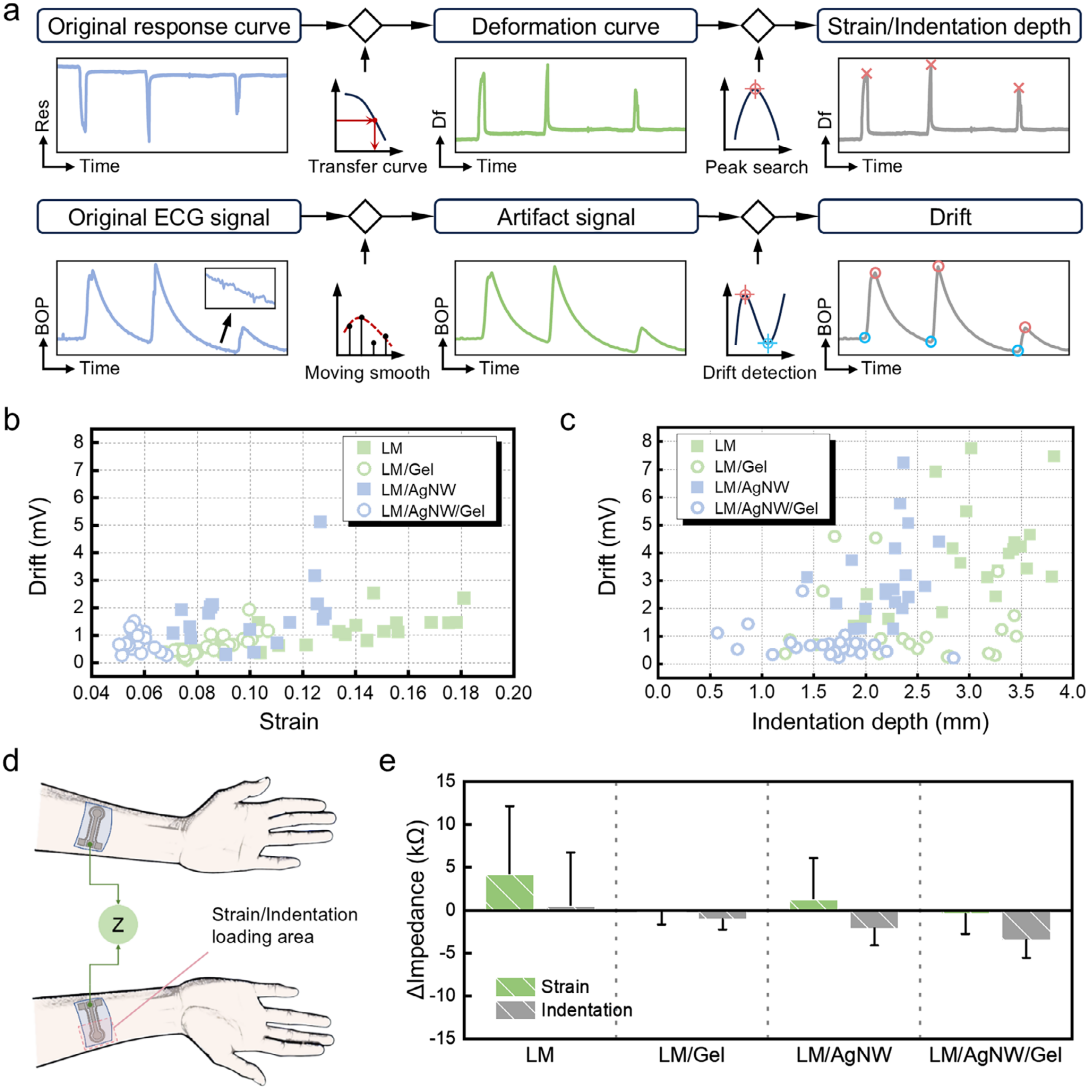
Quantitatively investigates the drift artifact alongside skin deformation. (a) The data processing workflow for the collected ECG signal and strain sensor data. (b) Drift in ECG signals versus skin strain scatter plot for four stretchable LM electrodes. (c) Drift in ECG signals versus indentation depth scatter plot for four stretchable LM electrodes. (d) Electrode placement and indentation/strain loading areas for skin‐electrode impedance testing. (e) Impedance changes under different skin deformation (strain ∼16%, indentation ∼3 mm) for four stretchable LM electrodes. Mean ± SD.

The study of pressure‐induced drift artifacts begins with the quantitative assessment of both skin deformation and drift artifacts in the ECG signal. Figure 2a illustrates the data processing workflow for the collected ECG signal and strain sensor data. The original resistance response curves measured by the strain sensor were converted into deformation curves based on the quantitative mapping functions established above, from which the peaks of strain/indentation depth were extracted. For the ECG (lead‐II) data collected from electrodes placed on both forearms, the artifact signal was estimated using a sliding average algorithm, and then the drift amplitude was quantitatively determined by analyzing drift onset and peak points.

The experimental results obtained from four stretchable LM electrodes are shown in Figure 2b,c. The results indicate that although the stretchable thin‐film electrodes exhibited minimal strain‐induced drift artifacts (less than 2 mV under 10% strain), they were highly susceptible to pressure‐induced drift artifacts (greater than 7 mV under a 3 mm indentation depth). Although the incorporation of conductive gel and silver nanowires mitigates the strain on the sensor and associated drift artifacts under identical external interference, this modification exhibits no significant effect on pressure‐induced drift. Experimental results involving more subjects using LM electrode further validate the reproducibility of this phenomenon (Figure S8). Meanwhile, although statistical analysis reveals inter‐subject variability among these subjects, significant differences in drift induced by pressure and strain still exist (Figure S9). Additionally, with the LM electrode attached to the forearm, we evaluated three daily scenarios: clothing pressure, weight lifting, and forearm rotation. Among these, both the clothing pressure and weight lifting tests, which involve mechanical pressure, induced noticeable drift noise in the ECG signals (Figures S10 and S11). In contrast, the forearm rotation test, which primarily involves strain, showed no drift phenomena significantly correlated with the motion (Figure S12).

Previous studies have predominantly employed the traditional skin‐electrode impedance model, attributing drift artifacts to changes in impedance between the skin and the electrode under external force [10, 20, 21]. However, our experimental findings have identified several contradiction in this model. Impedance characterization (Figure 2d) has performed using stretchable electrodes under identical conditions of strain (∼16%) and indentation (∼3 mm). The variations in skin‐electrode impedance (at 100 Hz) caused by strain and indentation are presented in Figure 2e (more details see Figure S13). However, a combined review with Figure 2b,c indicates that these impedance changes are not the dominant source of pressure‐induced drift artifacts. First, although indentation causes a smaller impedance change than strain in the LM electrode, it induces significantly greater signal drift. Second, the LM/AgNW/Gel electrode shows larger absolute impedance changes under both indentation and strain compared to the LM electrode, yet exhibits less drift. Third, despite the differing directions of impedance changes across electrodes, the direction of drift remains consistent.

To further evaluate the relationship between electrode–skin impedance changes and signal drift, statistical analyses were performed on data from six subjects under strain and indentation conditions. As shown in Figure S14, impedance changes did not differ significantly between the two conditions (*p* = 0.26233), whereas drift exhibited a statistically significant difference. The potential correlation between impedance change and drift was further investigated via a correlation analysis. For each subject and condition, the mean drift value (averaged over six repeated measurements) was paired with the corresponding absolute impedance change, yielding 12 paired observations (6 subjects × 2 conditions). The scatter plot (Figure S15) revealed a weak and non‐significant correlation (Pearson's r = 0.25499, *p* = 0.42381). Therefore, no significant correlation between these two variables can be established.

**FIGURE 3:**
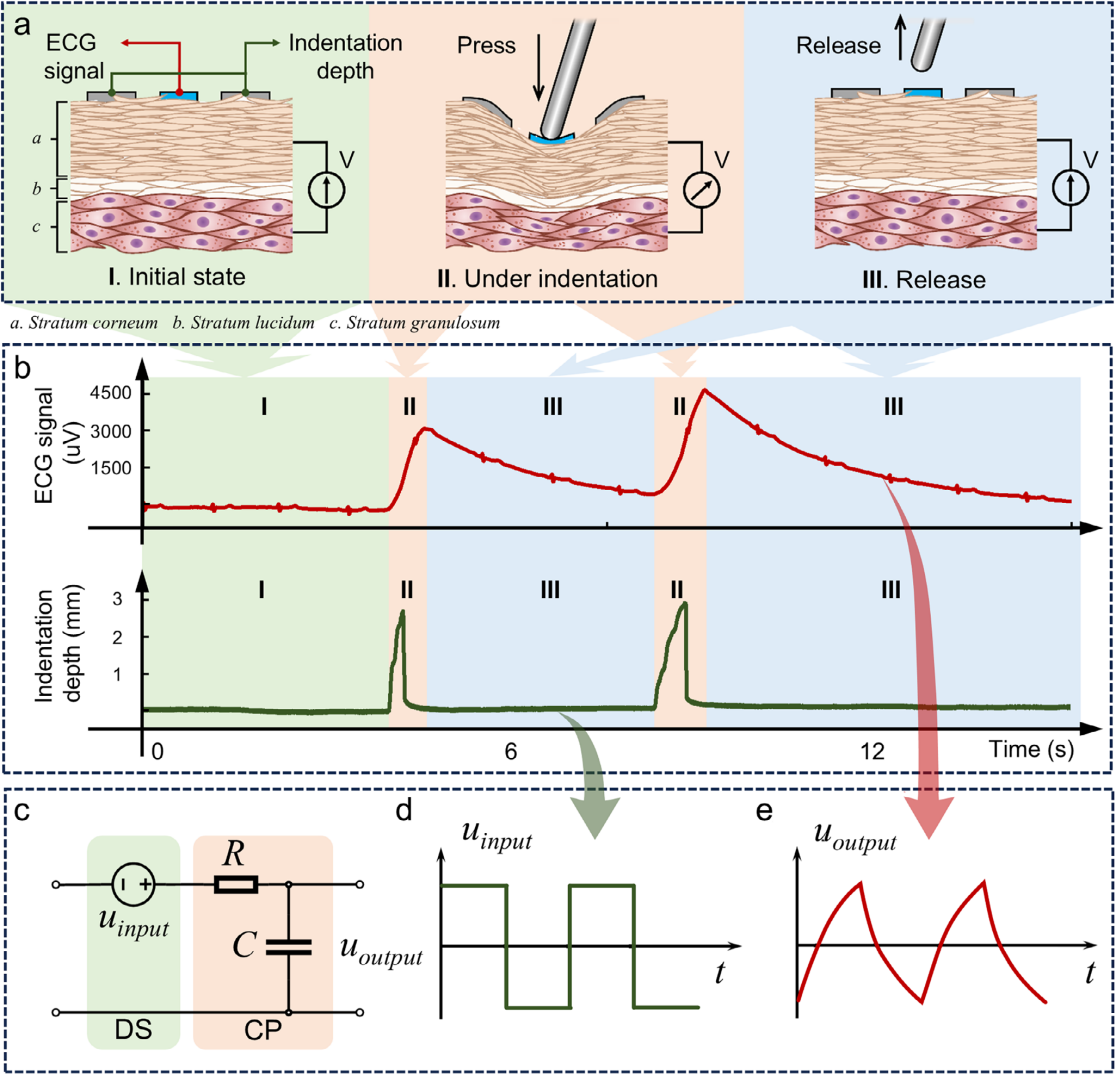
Principle explanation and analogy of pressure‐induced drift artifacts. (a) Skin potential change model. (b) The ECG signal with pressure‐induced drift artifacts and the indentation depth change curve collected by the annular strain sensor. (c) The skin potential model for pressure‐induced drifts, comprising a disturbance source (DS) and an RC coupling path (CP). (d) The disturbance source produces perturbation input induced by mechanical pressure. The perturbation input is generated from the disturbance source (induced by mechanical pressure). (e) The final drift output is formed as the perturbation input propagates through the RC coupling path.

The skin potential model can account for the occurrence of pressure‐induced drift phenomena. As shown in Figure 3a, indentation induces charge accumulation as well as the intrinsic potential difference (V) between the inner and outer layers of the skin [22, 23]. This transepidermal potential difference undergoes changes when the skin is subjected to pressure, leading to the observation of strongly correlated drift artifacts in ECG signals. To investigate this, two stretchable LM electrodes were placed on both forearms to simultaneously acquire ECG signals and indentation depth data. Following the procedure outlined in Figure 2a, indentation depth data were extracted and aligned with the ECG signals. The results depicted in Figure 3b show that the drift artifact exhibits a rapid rise followed by a slow decay, corresponding to the contraction and relaxation of the skin after indentation.

The pressure‐induced drift generation process is well represented by a model comprised by a disturbance source (DS) and a RC coupling path (CP) (Figure 3c). The perturbation source refers to the variation of the transepidermal potential difference induced by pressure [22, 24], generating a perturbation input similar to the pattern shown in Figure 3d. Given the negligible influence of minor impedance variations on drift (as addressed above), the RC parameters are idealized as constant. Upon passing through the RC path, the perturbation input generates the corresponding drift output depicted in Figure 3e.

### 2.3 Indentation‐Reconstruction Drift (IR‐drift) Method and Its Application

**FIGURE 4:**
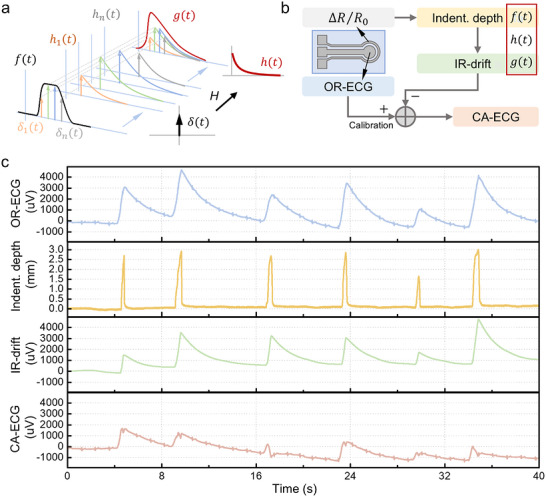
From indentation depth to pressure‐induced drift artifacts reconstruction. (a) Superposition and homogeneity of unit impulse response in linear time‐invariant systems. (b) Flow chart of obtaining the indentation‐reconstruction drift (IR‐drift) and calibrating the pressure‐induced drift artifact. (c) Raw and reconstructed waveforms (from top to bottom): (i) Original electrocardiogram (OR‐ECG) signals with pressure‐induced drift artifacts; (ii) Indentation depth data obtained from the annular strain sensor; (iii) IR‐drift signal reconstructed from the indentation depth data; (iv) Calibrated electrocardiogram signal (CA‐ECG) obtained by subtracting the IR drift from the OR‐ECG.

An indentation‐reconstruction drift (IR‐drift) method and calibration strategy based on indentation depth data collected by the strain sensor were developed. The reconstruction assumes that the process of the signal transformation, where indentation causes drift artifacts in ECG signals, satisfies the superposition and homogeneity properties of a linear time‐invariant (LTI) system. Figure 4a illustrates that within an LTI framework, any arbitrary input signal *f*(*t*) can be regarded as a series of unit impulse signals δ_*n*_(*t*) with different amplitudes. If the system's unit impulse response *h*(*t*) to δ(*t*) is known, its response *g*(*t*) to an arbitrary input *f*(*t*) can be expressed as the superposition of a series of impulse response components (*h_n_* (*t*)). Therefore, identifying the unit impulse response *h*(*t*) between the indentation depth and the drift in the ECG signal is key to the reconstruction process.

Inspired by the above‐mentioned similarity, we simulated the unit impulse response of the first‐order passive RC high‐pass filter shown in Figure S16. The unit impulse response between the indentation depth and the drift artifact is supposed as follows:

(1) $$h\;\left(t\right)=\alpha e^{-\beta t}$$

By gradient‐free, rule‐based iterative optimization of the parameters α and β, we maximized the similarity between the reconstructed drift artifact and the measured results (see Methods).

The calibration process is outlined in Figure 4b: under irregular external pressure disturbances, the raw resistance response data from the strain sensor were first converted into indentation depth data based on the sensor's quantitative mapping function; the IR‐drift signal was then reconstructed from the indentation depth data:

(2) $$g\;\left[n\right]=\sum_{k=0}^{N}f\left[k\right]h\left[n-k\right]\;$$

where *g*[*n*] represents the IR‐drift signal, *f*[*n*] is the indentation depth, and *h*[*n*] is the discrete impulse response function. Finally, IR‐drift was subtracted from the original ECG signal (OR‐ECG) acquired by the electrode to obtain the calibrated ECG signal (CA‐ECG).

Figure 4c presents the four key data waveforms from the calibration process. As shown in Figure 4c(i), the ECG signal exhibits pronounced drift artifacts under pressure interference, with amplitudes substantially larger than the effective waveform. Figure 4c(ii) illustrates that the indentation depth data, acquired and converted from the strain sensor, corresponds closely to the drift artifacts in the ECG signal. In Figure 4c(iii), the IR‐drift signal reconstructed from the indentation depth data demonstrates high similarity to the drift artifacts observed in the ECG. Finally, Figure 4c(iv) shows that directly subtracting the IR‐drift from the ECG signal results in a calibrated ECG in which the drift artifacts are markedly suppressed (reduction in peak value from 5 mV to 2 mV).

**FIGURE 5:**
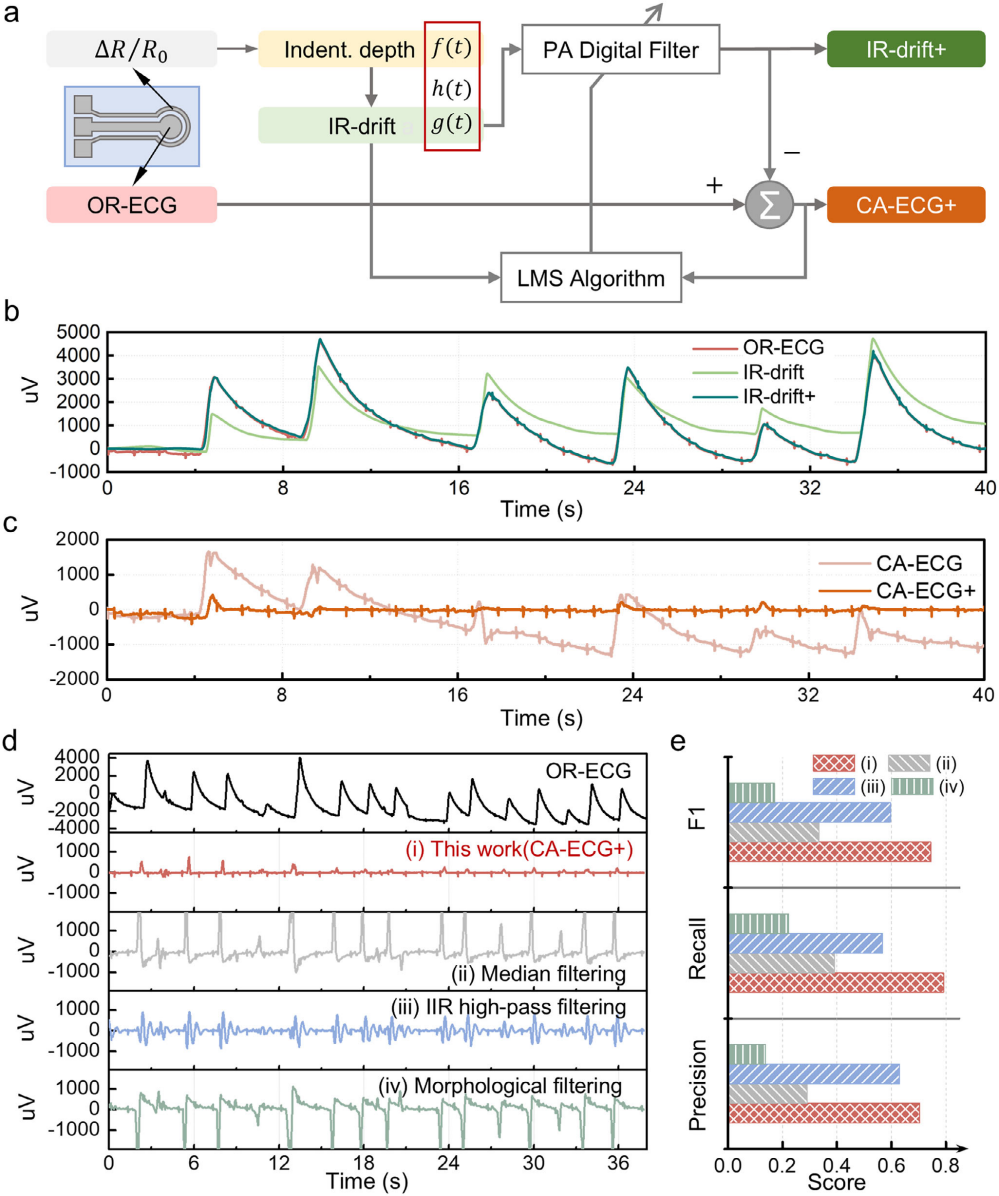
Drift calibration using IR‐drift and a self‐adaptive filter. (a) Adaptive filter based on least mean square (LMS) algorithm: (b) Comparison of OR‐ECG, IR‐drift, and IR‐drift+ (IR‐drift after the self‐adaptive filter). (c) Comparison of CA‐ECG and CA‐ECG+ (CA‐ECG after the adaptive filter). (d) Comparison of results between this work and other real‐time static filtering methods. (e) Comparison of R‐peak localization accuracy after different filtering.

Adaptive filtering method was employed to further enhance the calibration effect of the IR‐drift. Adaptive filtering minimizes the error signal by comparing the output signal with the desired signal and then adjusting the filter parameters through an adaptive algorithm [25, 26]. Therefore, selecting the correct input and desired signals is crucial for the effective adaptive filtering. As shown in Figure 5a, we introduced the IR‐drift as the input signal and the OR‐ECG signal as the desired signal into the adaptive filter. The least mean squares (LMS) algorithm was used to adaptively adjust the parameter adjustable (PA) digital filter to minimize the error signal (CA‐ECG) [27, 28].

The output signal of the PA digital filter, IR‐drift+, represents the IR‐drift more closely aligned with that in the OR‐ECG signal (Figure 5b). The final/target output of the adaptive filter is CA‐ECG+, which is the ECG signal after subtracting IR‐drift+. Figure 5c demonstrates the effectiveness of the method. Directly subtracting IR‐drift from OR‐ECG results in CA‐ECG, which still exhibits significant drift amplitude (approximately 2 mV). After adaptive filtering, the subtraction of IR‐drift+ from OR‐ECG yields CA‐ECG+, which exhibits a much smaller baseline drift (< 0.5 mV). Figure 5d compares the calibration effect achieved by our method with traditional real‐time static filtering methods, such as median filtering and IIR high‐pass filtering. Our method shows a 50% improvement (calculated by the drift amplitude) in drift artifact suppression (real‐time calibration demonstration is shown in Movie S2). We employed the Pan and Tompkins (P&T) algorithm [29] to detect the R‐peak location of the four results in Figure 5d. The positioning accuracy results are shown in Figure 5e, and it shows that the P&T algorithm achieved the best results on the signal that has undergone our calibration method. Both recall and precision are improved, and the F1 score is improved by 0.149 compared to other methods. Moreover, our method is self‐calibrating, making it more effective than static real‐time filters in handling drift artifacts across different frequency domains (Figure S17 demonstrates the limitations of using an IIR high‐pass filter to address indentation‐induced drift artifacts in ECG).

## 3 Discussion

For the pressure‐induced skin potential change model, the possible physiological mechanism can be attributed to the asymmetric distribution of ions (such as Na+, K+) between the dead cell layer (stratum corneum) and the living cell layer (stratum granulosum and below). As shown in Figure S18, under physiological conditions, a stable ionic concentration gradient exists across the skin, with higher ion concentrations in deeper layers and lower concentrations in superficial layers. This gradient is maintained by a dynamic equilibrium between passive diffusion from viable layers toward the stratum corneum and active ion pumping in the reverse direction. The interface between these layers acts as a semipermeable membrane that permits passive ion diffusion while supporting active transport, thereby establishing a stable inside‐positive, outside‐negative transepidermal potential. Mechanical deformation of the skin primarily modulates the ionic permeability of this semipermeable structure, leading to rapid changes in transepidermal potential that form the physiological origin of the observed drift disturbance. This mechanism accounts for the stronger influence of indentation compared with macroscopic strain: the flattened cells of the stratum lucidum between the stratum corneum and stratum granulosum feature narrow intercellular channels that dominate passive ion transport [30]. Even mild indentation can significantly compress these channels and alter their effective permeability, yielding a much more pronounced effect than equivalent mechanical strain. It should be emphasized that the above explanation represents a mechanistic hypothesis rather than a definitive conclusion. We speculate that prolonged skin deformation may not only alter membrane permeability but also gradually affect active ion transport processes, a topic that warrants further systematic investigation in future work.

In the present work, liquid metal was used for both electrodes and strain sensors, simplifying fabrication and ensuring good device uniformity. However, the liquid metal strain sensor's response to skin deformation relies primarily on geometric deformation effects, limiting its sensitivity and linearity. Future optimization should prioritize strain sensor linearity, with enhanced sensitivity providing additional power‐saving benefits. Specifically, improved linearity will further enhance calibration accuracy: the skin potential model assumes a linear time‐invariant relationship between indentation and drift, making accurate indentation measurement critical. Higher sensor linearity improves resistance‐to‐indentation mapping precision, enabling more accurate drift reconstruction and overall calibration performance. In contrast, enhanced sensitivity has limited impact on calibration accuracy but reduces power consumption: while higher sensitivity increases resistance change under skin deformation, the calibration method's sequential mapping processes (resistance‐to‐indentation and indentation‐to‐drift reconstruction) offset response amplitude differences via adaptive parameter adjustment. Nevertheless, higher sensitivity lowers front‐end acquisition circuit resolution requirements, reducing device power consumption.

Regarding the scalability of this study in electromyography (EMG) applications, a critical difference still exists: the signal source of ECG is located far from the skin surface, and skin deformation does not affect cardiac activity. In contrast, skin deformation can readily alter the morphology and activation patterns of underlying subcutaneous muscles. Indeed, previous studies have demonstrated that external pressure can influence muscle activation [31, 32]. Therefore, for EMG applications, the model proposed in this study—which only accounts for transcutaneous potential interference along the signal transmission path—requires further optimization to incorporate changes in the signal source itself.

## 4 Conclusion

This work demonstrates the severe impact of pressure‐induced drift motion artifacts on signal quality using stretchable liquid metal thin‐film ECG electrodes, while highlighting the intimate relationship between these artifacts and the skin potential model. We further propose a method to reconstruct these artifacts using skin deformation data, which is then applied for real‐time calibration of ECG to suppress noise caused by irregular pressure interference. This work holds particular significance as it breaks the prevailing neglect of pressure interference in thin‐film ECG device studies. Besides, the proposed indentation‐reconstruction drift (IR‐drift) algorithm offers insights for achieving more robust ECG signal acquisition. In the future, this work can be further extended through algorithm innovation and system integration validation [33, 34].

## 5 Experimental Section

### 5.1 Materials Preparation

Pressure‐sensitive adhesive coating was purchased from 3 M company (SP‐7533, USA). Smooth‐On (USA) Ecoflex00‐30 was used as the temporary test substrate. The PU fiber film was prepared using an electrospinning machine (Beijing Yongkang Leye Technology Development Co., Ltd, China). The specific preparation parameters are as follows: the surface of the drum receiver (10 cm diameter and 20 cm height) is covered with aluminum foil, the speed is 20 r/min, the injection speed of the PU solution (5 mL KDL syringe) is 0.2 mm/min, the needle translation distance is 20 cm, the translation speed is 20 cm/min, the distance between the needle and the drum surface is 16 cm, and the voltage is 15 kV, the needle size is 21G and the needle length is 13 mm. The preparation time is 90 min. The medical conductive gel was purchased from Letai Medical Technology Co., Ltd (Suzhou, China). The LM stretchable conductive ink was purchased from Rouzhi Technology Co., Ltd (Ela‐1000 M, Beijing, China). The water‐based silver nanowire (AgNW) solution (diameter 100–150 nm, 10 mg/mL) was purchased from XFNANO (Nanjing, China) Materials Technology Corporation. The commercial gel electrode used in this article was purchased from Hangzhou Xunda Radio Equipment Co., Ltd. China, and it meets the medical devices standards of the National Medical Products Administration of China.

### 5.2 Fabrication Process of the Electrode

The electrode was fabricated via a scalable screen‐printing process. The detailed fabrication workflow is shown in Figure S2. The process is divided into two main steps: fabrication of the three upper layers (PU, adhesive, and sensing) and fabrication of the bottom PSA layer with laser‐cut openings for the electrodes. Figure S2(i–iv) outlines the fabrication process from the top layer to the sensing layer. Initially, the sensing layer containing electrodes and strain sensors were created on a polyethylene terephthalate (PET) release film using a custom screen‐printed stencil and LM paste (i). Following oven heating to remove the solvent, a PSA coating was applied to the sensing layer surface using a 100‐µm‐thick film applicator to form the middle adhesive layer (ii). To prevent bubble formation in the final middle layer due to heating, the PSA was cured at room temperature before a pre‐fabricated PU fiber film was laminated onto the adhesive layer (iii). The resulting layers was then peeled off the PET release film (iv). Figure S2(v–vi) describe the preparation of the bottom PSA layer with the hollow pattern. A PSA layer was directly coated onto the PET release film using a 100‐µm‐thick film applicator (v). After room temperature curing, a laser etching system (Universal Laser, USA, VLS2.30DT‐SYS) was utilized to create the hollow pattern (vi). Figure S2(vii) illustrates the alignment and combination of the three upper layers with the bottom layer to form the complete device. The resulting device retained the PET release film as a temporary protective substrate, which could be removed before use.

### 5.3 Measurement of Device Thickness and Young's Modulus

A thickness gauge (Resolution 1 µm, SYNTEK, Germany) was used to measure the device thickness. Randomly select 5 points from three samples for measurement and take the average value as the thickness. Young's modulus measurement was carried out by AGS‐X (Shimadzu), the measured curve and size of the samples are shown in Figure S1.

### 5.4 Data Recording

The ECG signal is recorded by a self‐developed acquisition circuit with a sampling rate of 250 Hz. The self‐developed acquisition circuit consists of an analog front‐end chip (ADS1298) with a 24‐bit analog‐to‐digital converter (ADC), a control chip (STM32F103C8T6), and a power module. The data is sent to the computer through a serial port for storage. The resistance of the strain sensor is measured by DM3068 Digital Multimeter (Rigol Technologies, China). Recording instructions can be found in Figure S7. The sampling rate of the strain signal is 500 Hz, and aligning these data is achieved by discarding the sampling point at the computer.

### 5.5 Fourier Fitting

The Fourier numerical fitting was conducted using the Curve Fitting tool available in MATLAB R2016a. For the strain testing data presented in Figure 1d, a second‐order Fourier fitting function was employed:

(3) $$f\;\left(t\right)=a_{0}+a_{1}\cos\left(\omega t\right)+b_{1}\sin\left(\omega t\right)+a_{2}\cos\left(2\omega t\right)+b_{2}\sin\left(2\omega t\right)$$

where *a*_0_ =   − 0.01963 (− 0.01964, −0.01962),   *a*_1_ =  0.0168 (0.01679, 0.01681),  *b*_1_ =   − 0.0006075 (− 0.0006462, 0.0005687),  *a*_2_ =  0.003106 (0.003098, 0.003114),  *b*_2_ =   − 0.001137 (− 0.001146, −0.001128), ω  =  13.2 (13.18, 13.21).

For the pressure testing data presented in Figure 1e, a first‐order Fourier fitting function was utilized:

(4) $$f\;\left(t\right)=a_{0}+a_{1}\cos\left(\omega t\right)+b_{1}\sin\left(\omega t\right)$$

where *a*_0_ =   − 0.03548 (− 0.03557, −0.0354),  *a*_1_ =  0.03501 (0.03492, 0.03511),  *b*_1_ =  0.005411 (0.005349, 0.005473), ω  =  0.3008 (0.3001, 0.3016).

### 5.6 Skin‐Electrode Impedance Measurement

Different electrodes were attached to the inside of the right forearm as shown in Figure 2d. VICTOR 4092E bench bridge tester (Shenzhen Yisheng Victory Technology Corporation, China) was used to measure the skin‐electrode impedance. A fixed strain of ∼16% was applied to one of the electrodes to measure the impedance change before and after the strain. The quantitative application of strain relies on positioning stickers attached to the skin. A 100 g weight is used as a fixed source of pressure (∼3 mm indentation). The experimental procedure is as follows: first, measure the skin‐electrode impedance without applying strain, then apply 16% skin strain and measure the skin‐electrode impedance again. Following that, apply a pressure of a 100 g weight to the electrode to measure the skin‐electrode impedance.

### 5.7 Drift Artifacts in Electrocardiogram and Skin Deformation Measurement

Three subjects were asked to wear stretchable LM thin‐film electrodes on their left and right forearm, one experimenter wore gloves and randomly applied strain around the electrode on the right forearm while recording the original ECG signal and strain sensor data. In addition, the experimenter will also randomly apply pressure to the electrode using a stainless steel hemisphere, and simultaneously record the original ECG signals and indentation data from the strain sensor. When conducting experiments requiring precise control of applied strain and indentation depth, variable regulation is realized by marking scale lengths on the skin surface and adopting mechanical compression (AGS‐X, Shimadzu, Japan)).

### 5.8 Using Impulse Response Function to Calculate IR‐Drift Signal in Real‐Time

The recursive formula for the real‐time indentation‐reconstructed drift signal calculation can be obtained using a linear constant coefficient difference equation:

(5) $$g\;\left[n\right]=e^{-b}\;g\left[n-1\right]+f\left[n\right]h\left[0\right]$$

### 5.9 Optimizing the Parameters *α* and *β* in the Unit Impulse Response Function

There are two ways to obtain optimized parameters *α* and *β*: manual and automatic.

(i) Manual optimization: because the indentation‐reconstructed signal will be processed by adaptive filtering to align with the true drift artifact in the ECG. The manual approach focuses on obtaining an IR‐drift signal similar to the overall trend. The process consists of the following steps: (1) Select an ECG signal with a corresponding indentation depth data, ensuring the inclusion of at least three distinct signal responses caused by pressure. (2) Randomly assign initial values to *α* and *β* in the unit impulse response function and use Equation (5) to generate the reconstructed signal. (3) Compare the IR‐drift signal to the drift artifacts in the ECG signal, adjusting *α* to modify peak fluctuations and *β* to adjust the attenuation rate of the drift in the reconstructed signal. (4) Repeat adjustments until the reconstructed signal closely matches the ECG baseline visually. Once this is achieved, *α* and *β* are considered optimized parameters, and the corresponding unit impulse response function is applied.

(ii) Automatic optimization: The first and second steps of automatic optimization are the same as manual optimization. Starting from the third step, automatic optimization sets a quantitative optimization objective to automatically update *α* and *β*. The steps are as follows: extract the peak‐to‐peak value *P_target_* of the maximum baseline drift and the time *T_target_* of baseline drift attenuation from the ECG signal, extract the peak‐to‐peak value *P_current_* of the maximum drift and the time *T_current_* of drift attenuation from the IR‐drift signal and update the parameters according to the following conditions:

(6) $$\left\{\begin{array}{c} \;\alpha_{new}=\alpha_{last}-u_{\alpha },ifP_{current}-P_{target}>P_{threshold}\\ \;\alpha_{new}=\alpha_{last}+u_{\alpha },ifP_{current}-P_{target}<-P_{threshold} \end{array}\right.$$

When |*P_current_* − *P_target_* | < *P_threshold_*, stop and get the optimized parameters *α*. *P_threshold_* is the optimize termination threshold.

(7) $$\left\{\begin{array}{c} \;\beta_{new}=\beta_{last}+u_{\beta },ifT_{current}-T_{target}>T_{threshold}\\ \;\beta_{new}=\beta_{last}-u_{\beta },ifT_{current}-T_{target}<-T_{threshold} \end{array}\right.$$

When |*T_current_* − *T_target_* | < *T_threshold_*, stop and get the optimized parameters *β*. *T_threshold_* is the optimize termination threshold. Where *u*_α_ and *u*_β_ are the learning rates for *α* and *β*, respectively. A more detailed description of the automatic optimization method can be found in the supplementary material (Figure S19).

### 5.10 Least Mean Squares (LMS) Adaptive Filtering

The implementation of LMS adaptive filtering here relies on a parameter adjustable (PA) digital filter and an LMS algorithm for parameter updates. The length of the PA digital filter is set to N, and the weight parameters W⃗Δ=[w1,w2,…,wN]T are randomly initialized. Input signal X⃗Δ=[x1,x2,…xn]T passes through the PA digital filter to obtain output signal Y⃗Δ=[y1,y2,…yn]T:

(8) $$y\;\left[n\right]=\sum_{k=1}^{N}w\left[k\right]x\left[n-k+1\right]\;$$

The filtering error *e*[*n*] are calculated by output signal *y*[*n*] and desired signal *d*[*n*]:

(9) $$e\;\left[n\right]=d\left[n\right]-y\left[n\right]$$

Under the mean square error (MSE) criterion, the optimal filter weights minimize the cost function:

(10) JW⃗Δ=minEen2

LMS algorithm continually attempts to reduce the MSE by updating the weight vector, at each time instant, as

(11) $$\overset{\to }{\underset{j+1}{W}}=\overset{\to }{\underset{j}{W}}+2\mu e\left[n\right]\overset{\to }{\underset{n-N+1∼n}{X}}$$

where $\overset{\to }{\underset{n-N+1∼n}{X}}={\left[x_{n-N+1},x_{n-N+2},\text{…}x_{n},\right]}^{T}\;$, μ is the learning rate.

## Author Contributions

D.L. and Z.G.X. made equal contributions and are co‐first authors. The manuscript was written through the contributions of all authors. All authors have given approval to the final version of the manuscript. Conceptualization: T.L.R., Y.Y., L.Q.T. Methodology: D.L., Z.G.X., Z.K.C., S.Y.X. Investigation: D.L., Z.G.X., Z.K.C., S.Y.X. Visualization: D.L. Supervision: T.L.R., Y.Y., L.Q.T., X.M.W., H.F.L. Writing‐ original draft: D.L., Z.G.X. Writing‐ review & editing: D.L., Z.G.X., H.F.L., T.L.R. Funding acquisition: T.L.R. Experiment: D.L., Z.G.X., Z.K.C., S.Y.X. J.Y.C., S.Y.W., Z.X.W., Y.K.L., and J.J.Y.

## Funding

This work was supported by the National Natural Science Foundation of China (Grant Nos. 92580209, U25A6021), the Scientific and Technological Innovation Project of China Academy of Chinese Medical Sciences (Grant Nos. ZN2023A01, CI2023C002YG), and the Institute for Intelligent Healthcare at Tsinghua University(Grant No. 2023ZLB001).

## Ethical Statement

The study protocol was thoroughly reviewed and approved by the ethical committee of Tsinghua University (approval no. 20220227) and was conducted in accordance with the Declaration of Helsinki. Informed consent was obtained from all participants for all experiments.

## Conflicts of Interest

The authors declare no conflicts of interest.

## Supporting information




**Supporting File 1**: advs76002‐sup‐0001‐SuppMat.docx.

**Supporting File 2**: advs76002‐sup‐0002‐MovieS1.mp4.

**Supporting File 3**: advs76002‐sup‐0003‐MovieS2.mp4.

## Data Availability

The data that support the findings of this study are available from the corresponding author upon reasonable request.
