# Supplementary material for: Pressure‐Induced Drift Artifacts in Stretchable Liquid Metal ThinFilm Electrocardiogram Electrodes
Source: Adv Sci (Weinh). 2026 Jul 2;13(49):e76002. doi: 10.1002/advs.76002 (PMC13336560; doi:10.1002/advs.76002)
Supplement: Supplementary file 1 [file ADVS-13-e76002-s003.docx]

Supplementary Materials for

**Pressure-Induced Drift Artifacts in Stretchable Liquid Metal Thin-film Electrocardiogram Electrodes**

Ding Li, Zi-Gan Xu, Zhi-Kang Chen, Shuo-Yan Xu, Jia-Yi Cui, Si-Yuan Wo, Zi-Xu Wang, Yi-Kun Liu, Jia-Ju Yin, Xiao-Ming Wu, Hou-Fang Liu, Xiao-Ming Wu, Liu-Qi Tao, Yi Yang, Tian-Ling Ren*.*

*Corresponding author. Email: rentl@tsinghua.edu.cn (T.L.R.); yiyang@tsinghua.edu.cn (Y.Y.); taoluqi@tsinghua.edu.cn (L.Q.T.)

**This PDF file includes:**

Figs. S1 to S19

**Other Supplementary Materials for this manuscript include the following:**

Movies S1 to S2

Fig. S1. Structural dimensions of the stretchable LM thin-film electrode with an annular LM strain sensor.

Fig. S2. Flowchart of device fabrication based on screen printing process.

Fig. S3. Tensile testing curve of stretchable LM electrodes. Tensile speed 1 mm/s.

Fig. S4. Preparation and structure of four different stretchable LM electrodes utilizing different surface modification methods. (a) LM electrodes, (b) LM electrodes coated with medical conductive gel, (c) LM electrodes with deposited silver nanowires, and (d) LM electrodes that incorporate both silver nanowires and medical conductive gel.

Fig. S5. The skin-electrode impedance and intrinsic impedance of the four stretchable LM electrodes and the commercial electrode. (a) Skin-electrode impedance vs frequency. (b) Intrinsic impedance vs frequency. These measurements were performed using a KEYSIGHT E4980AL LCR digital bridge over a frequency range of 20 Hz to 10 kHz, with a 1 V sinusoidal test signal.

Fig. S6. Cyclic compression response test results under different measuring ranges.

Fig. S7. Instructions for the signal acquisition system of the test platform.

Fig. S8. Drift in ECG signals versus skin strain/indentation depth scatter plot with stretchable LM electrodes for six subjects.

Fig. S9. Multi-factor box plots of drift data categorized by skin deformation mode and subject. Boxes span the 25th to 75th percentiles, and whiskers extend to ±1.5 standard deviations from the mean. Within each subject, significance was determined by one-way ANOVA with Fisher’s LSD test. Across subjects, statistical significance was assessed using two-way ANOVA with Tukey’s post hoc test.

Fig. S10. ECG and strain sensor response waveforms recorded by repeatedly applying clothing pressure to three subjects. Method and description: The clothing pressure test on the forearm was designed to simulate the constriction from a shirt cuff. To ensure consistency, we prepared an elastic band with an original length of ~23 cm for all three subjects. At the start of the test, the band was worn around the subject’s wrist. During testing, the subject repeatedly pulled the band up to the electrode site on the forearm, maintained the constriction for approximately 0.5 s, and then returned it to the wrist. Although inter-subject variability resulted in morphological differences in drift amplitude and sensor response, clothing pressure induced significant drift noise in all three subjects.

Fig. S11. ECG and strain sensor response waveforms recorded by repeatedly applying 1kg carton pressure to three subjects. Method and description: The weight lifting test simulated routine object handling. The subject sat upright, with the palm facing upward and the forearm held perpendicular to the torso. A 600×600×3 mm acrylic sheet was placed on the palm and forearm, supported stably by both. During the test, the experimenter repeatedly placed a 1 kg cardboard box onto the sheet, held it for approximately 0.5 s, and then removed it. Despite some variation attributable to individual subject conditions, weight lifting introduced drift noise in all three subjects.

Fig. S12. ECG and strain sensor response waveforms recorded when three subjects rotated their forearms. Method and description: The forearm rotation test aimed to mimic daily strain interference. Subjects were instructed to rotate the forearm to flip the palm through 180° (alternating between palm-up and palm-down positions). No drift noise clearly correlated with the motion was observed in Subjects 1 and 2. In Subject 3, only two drift events were recorded—likely due to changes in device contact caused by the spring pollen allergic skin condition—because their timing did not align with the forearm rotation motion.

Fig. S13. Skin-electrode impedance versus frequency curves under different skin deformation conditions for four stretchable LM electrodes.

Fig. S14. Impedance variation and drift under strain (~16%) and indentation (~3 mm) conditions. Mean ± SEM. Kruskal Wallis test.

Fig. S15. Correlation between impedance variation and drift (r = 0.25499, p = 0.42381).

Fig. S16. Unit impulse response of the first-order RC high-pass filter. $h(t)$ is the unit impulse response function.

Fig. S17. Using an IIR high-pass filter to address pressure-induced drift artifact in ECG signals. (a) An ECG signal with pressure-induced drift artifacts. (b) The ECG signal processed by a IIR digital filter (Butterworth) with cut-off frequency of 0.5 Hz. (c) The ECG signal processed by a IIR digital filter (Butterworth) with cut-off frequency of 1 Hz.

Fig. S18. Illustrations of the possible physiological mechanism underlying the skin potential change model.

Fig. S19. Calibration and adaptive filtering pipeline for indentation-induced ECG drift correction.

The automatic optimization follows a rule-based iterative scheme, which operates in the calibration stage before the main signal collection stage:

1. **Data acquisition and preprocessing**: Synchronous ECG and indentation data are acquired, and the indentation signal is used to reconstruct the IR-drift response based on the preset model.
2. **Feature extraction**: From the measured ECG signal, we extract the peak-to-peak amplitude of the maximum baseline drift (*P*_target_) and the drift half-attenuation time (*T*_target_) as reference targets. From the reconstructed IR-drift signal, we extract the corresponding current values (*P*_current_ and *T*_current_).
3. **Iterative parameter update**:

1) Parameter *α* is adjusted using a predefined learning rate *u_α_* to minimize the difference between *P*_current_ and *P*_target_. If |*P*_current_ - *P*_target_| is smaller than the termination threshold *P*_thr_, the iteration stops and the optimized *α* is obtained.

2) Parameter *β* is adjusted using a predefined learning rate *u_β_* to minimize the difference between *T*_current_ and *T*_target_. If |*T*_current_ - *T*_target_| is smaller than the termination threshold *T*_thr_, the iteration stops and the optimized *β* is obtained.

4. **Calibrated model deployment**: The optimized *α* and *β* are then fixed and used in the subsequent collection stage, where real-time IR-drift reconstruction and adaptive filtering are performed to produce the calibrated ECG signal.

This gradient-free, rule-based iterative optimization enables automatic, subject-specific calibration without manual intervention. The flowchart clearly maps each step of the calibration loop to the corresponding signal-domain effects shown on the right-down of this figure: the iterative updates progressively adjust the amplitude and decay characteristics of the IR-drift response (blue curves) to match the measured ECG drift (red curve), until the matching criteria are met.
